# Synergism between Prior *Anisakis simplex* Infections and Intake of NSAIDs, on the Risk of Upper Digestive Bleeding: A Case-Control Study

**DOI:** 10.1371/journal.pntd.0001214

**Published:** 2011-06-28

**Authors:** Florencio M. Ubeira, Ana M. Anadón, Angel Salgado, Alfonso Carvajal, Sara Ortega, Carmelo Aguirre, María José López-Goikoetxea, Luisa Ibanez, Adolfo Figueiras

**Affiliations:** 1 Department of Microbiology and Parasitology, Faculty of Pharmacy, University of Santiago de Compostela, Santiago de Compostela, Spain; 2 Consortium for Biomedical Research in Epidemiology and Public Health (CIBER en Epidemiología y Salud Pública - CIBERESP), Santiago de Compostela, Spain; 3 Department of Preventive Medicine and Public Health, University of Santiago de Compostela, Santiago de Compostela, Spain; 4 Department of Pharmacology, Faculty of Medicine, University of Valladolid, Valladolid, Spain; 5 Pharmacosurveillance Unit, Galdakao-Usansolo Hospital, Galdakao, Spain; 6 Microbiology Service, Galdakao-Usansolo Hospital, Galdakao, Spain; 7 Department of Pharmacology, Therapeutics and Toxicology, Autonomous University, Catalonian Institute of Pharmacology, Clinical Pharmacology Service, Vall d'Hebron University Teaching Hospital, Barcelona, Spain; National Institutes of Health, United States of America

## Abstract

**Background:**

The aim of this study was to investigate the relationship between prior *Anisakis* infections and upper gastrointestinal bleeding (UGIB), and its interaction with non-steroidal anti-inflammatory drug (NSAID) intake.

**Methods/Principal Findings:**

We conducted a hospital-based case-control study covering 215 UGIB cases and 650 controls. Odds ratios (ORs) with their confidence intervals (95% CIs) were calculated, as well as the ratio of the combined effects to the sum of the separate effects of *Anisakis* allergic sensitization and NSAIDs intake. Prior *Anisakis* infections were revealed by the presence of anti-*Anisakis* IgE antibodies specific to the recombinant Ani s 1 and Ani s 7 allergens used as the targets in indirect ELISA. Prior *Anisakis* infections (OR 1.74 [95% CI: 1.10 to 2.75]) and the intake of NSAIDs (OR 6.63 [95% CI: 4.21 to 10.43]) increased the risk of bleeding. Simultaneous NSAIDs intake and *Anisakis* allergic sensitization increased the risk of UGIB 14-fold (OR = 14.46 [95% CI: 6.08 to 34.40]). This interaction was additive, with a synergistic index of 3.01 (95% CI: 1.18–7.71).

**Conclusions:**

Prior *Anisakis* infection is an independent risk factor for UGIB, and the joint effect with NSAIDs is 3 times higher than the sum of their individual effects.

## Author Summary

*Anisakiasis* is a worldwide re-emerging disease produced by the consumption of raw, lightly cooked, smoked or marinated fish containing live *Anisakis* larvae. In acute anisakiasis, mucosal lesions generated by the larvae may provoke upper gastrointestinal bleeding (UGIB). However, the effect of past unnoticed *Anisakis* infections as a risk factor for UGIB, and a possible synergism with other risk factors such as NSAIDs intake, have never been investigated. In this case-control study we observed that: i) prior *Anisakis* infections and NSAIDs intake are two independent risk factors for UGIB, and ii) that both risk factors act synergistically to the extent that their joint effect is 3 times higher than the sum of their individual effects. We concluded that, in countries where *Anisakis* infections are frequent, it would be wise to determine parasite-specific IgE antibodies and to conduct a closer follow-up of patients who consume raw or lightly cooked fish and who are prescribed NSAIDs for long periods.

## Introduction

Upper gastrointestinal bleeding (UGIB) is a relatively frequent and potentially lethal multicausal medical emergency [1]. Gastric and duodenal ulcers are a major cause of UGIB, and bleeding from these lesions is frequently related to intake of non-steroidal anti-inflammatory drugs (NSAIDs) [2]. In countries where *Anisakis* infections are frequent, acute infections by this parasite may also provoke UGIB [3].

Anisakiasis is a worldwide re-emerging disease produced by the consumption of raw, lightly cooked, smoked or marinated fish containing the infective larvae of the *Anisakis* genus [4], [5]. Most human cases of anisakiasis have been reported in Japan [6], [7], but there has been an increase in the frequency of reports of *Anisakis* infections in other parts of the world, such as Europe [8], [9], the USA, [10], [11] and Canada [12].

Depending on the site of infection and the predominant clinical symptoms, acute infections by *Anisakis* can be classified as gastric anisakiasis, gastro-allergic anisakiasis, and intestinal anisakiasis. In gastric and intestinal anisakiasis, severe gastric or abdominal symptoms predominate, while in gastro-allergic anisakiasis, allergic symptoms ranging from mild urticaria to anaphylactic shock are more important [13], [14]. However, recent evidence from seroepidemiologic studies undertaken in Spain indicates that the great majority of human cases of anisakiasis are asymptomatic, and that the prevalence of disease in different Spanish regions may range from a minimum of 0.4% [5] to more than 10% of the population [15], [16].

In comparison with the healthy population, a high seroprevalence of anti-*Anisakis* antibodies has been reported in patients with GI bleeding [17]. However, the relevance of prior *Anisakis* infections as a risk factor for UGIB and its possible interaction with NSAID intake have never been investigated. We now report the results of a case–control study, which sought to determine the risk of UGIB associated with prior *Anisakis simplex* infections and any potential interaction with NSAID intake.

## Methods

### Patients

We based our study on data provided by a wider, multicenter, incident case-control study, which sought to analyze the influence of environmental and genetic risk factors on UGIB (primary study). Three Spanish hospitals (Complejo Hospitalario Universitario de Santiago de Compostela, Galicia; Hospital Clínico Universitario de Valladolid, Castilla-León; and Hospitales de Galdakao-Usansolo/Basurto, Basque Country) that had stored serum samples for *Anisakis* determinations were included in the study. We defined cases as any patient admitted in the period 2003–2006 with primary diagnosis of UGIB and subsequent endoscopic diagnosis of duodenal or gastric ulcer, acute lesions of the gastric mucosa, erosive duodenitis or mixed lesions. To ensure that cases and controls come from the same source population, all patients were recruited from the same hospitals [18]. For each case, we selected 3 controls, matched by sex, age (±5 years), hospital and point in time. To avoid selection of controls being associated with exposure to NSAIDs, the controls were recruited from among patients in preoperative care for scheduled surgical interventions for non-painful processes such as cataracts, inguinal or umbilical hernias, and prostate adenomas. The enrolment criteria of the primary study (cases and controls) excluded patients who, at the starting date, had a history of cancer, coagulopathy, Mallory-Weiss syndrome or esophageal varices, and subjects who were not resident in the study area.

#### Anti-*Anisakis* IgE determinations

Prior *Anisakis* infections were detected by investigating patient's sera for the presence of anti-*Anisakis* IgE antibodies to the Ani s 1 [19] and Ani s 7 [20] allergens, which are secretory antigens only produced by the parasite while the infecting larvae remain alive [21]. The IgE determinations were by indirect ELISA as previously described [22]. Briefly, wells in columns 1, 4, 7, and 10 of the 96-well microtiter plates (Greiner Bio-One, Frickenhausen, Germany) were filled with 100 µl of phosphate-buffered saline (PBS) containing rAni s 1 at a concentration of 5 µg/ml, and wells in columns 2, 5, 8, and 11 were filled with 100 µl of 0.1 M Tris buffer, pH 10.5, containing 0.6 µg/ml of t-Ani s 7. The wells in the remaining columns (controls) were filled with PBS alone. After incubation of the plates at 4°C overnight and washing with Tris-buffered saline containing 0.2% Tween 20 (TBS-T), the nonreactive sites were blocked with 200 µl of TBS-T containing 1% of skimmed dry milk (TBS-T1) for 2 h at 37°C. Later, 100 µl of undiluted serum was added to each well and incubated for 2 h at 37°C. After a washing step, specific IgE antibodies were detected by incubation first with 100 µl of a mouse IgE anti-human mAb (Ingenasa, Madrid, Spain; dilution 1∶5000 in TBS-T1) labeled with fluorescein isothiocyanate (FITC) and afterwards with 100 µl of peroxidase-conjugated rabbit anti-FITC Ig (Abcam, Cambridge, England; 1∶5000 in TBS-T1). Optical densities (ODs) at 492 nm were calculated by subtracting the OD value produced by the same serum in the absence of antigen. Patients displaying specific IgE antibodies to either of these allergens were classified as positive. All IgE determinations were performed in duplicate in a single laboratory.

#### Anti-*Helicobacter pylori* IgG determinations

Anti-*H. pylori* IgG antibodies were determined in human serum, using a commercially available ELISA kit (Trinity Biotech Captia, Co. Wicklaw, Ireland) in accordance with the manufacturer's protocol. The choice of this method was based on previous studies, which reported that serological methods for *H. pylori* determinations are not influenced by UGIB [23]. For statistical analysis purposes, dubious results were deemed negative.

### Variable definitions

Qualified, purpose-trained health staff interviewed cases and controls, after first obtaining written informed consent from the subjects. Pharmacologic anamnesis was comprehensive. Patients were first asked about any medications consumed during the two months prior to admission, and were then presented with a list of symptoms usually associated with NSAID consumption and asked whether they had taken any medication for any of these. Finally, patients who failed to remember the name of any medication were later telephoned at home to enable them to provide the name.

We defined an NSAID consumer as any subject shown by pharmacological anamnesis to have consumed some medication belonging to this therapeutic group in the week preceding the index date. Subjects taking Acetylsalicylic acid at doses of less than 0.125 g/day (considered to be antiaggregant) were not considered as NSAIDs consumers. Also, NSAIDs and corticoids administered by ophthalmic, dermal or rectal route were not deemed to be exposures.

We calculated a case index date, based on disease course and symptom-onset dates. For controls, the index date was the date of the interview. For cases, the consumption of medications between index and interview dates was not taken into account. Finally, we reviewed and assessed endoscopy reports of all cases according to whether or not they described detection of *Anisakis* larvae in the stomach or duodenum.

### Statistical calculations

We calculated odds ratios (ORs) and their adjusted confidence intervals (CIs) using hierarchical logistic model through a generalized linear mixed model [24]. For the purpose of constructing such models, patients were taken as level one, strata (each case and their matched controls) as level two, and hospitals as level three. In the estimation of the models we used the *lmer* function, implemented in the context of the lme4 R package (version 2.7.2) [25]. To construct these models, a bivariate analysis of each independent variable was performed, and variables with 0.2 in the bivariate analysis were then included in the multivariate analysis. Independent variables with the highest level of statistical significance were successively eliminated from the original model, provided that the coefficients of the principal variables of exposure changed by no more than 10% and Schwarz's Bayesian Information Criterion (BIC) improved [26]. The confidence intervals of the interaction terms were calculated using the method proposed by Figueiras et al. [27]. The results of the generalized linear mixed model were validated by comparing them against results from comparable models obtained by running conditional logistic regression. We calculated the ratio of the combined effects to the sum of the separate effects of *Anisakis* and NSAID (S) (along with its 95% confidence interval) [28] as a measure of additive interaction, [18] since S has been shown to be the most reliable measure of additive interaction when adjusting for confounding [29].

### Ethics statement

The study protocol was approved by the following ethics committees: i) Comité Etico de Investigación Clínica de Galicia; ii) Comité Etico de Investigación Clínica de la Universidad de Valladolid; and iii) Comité Etico de Investigación Clínica del Hospital de Galdakao-Usansolo. All cases and controls were required to give written informed consent, and where such approval was not forthcoming, the subject concerned was excluded from the study.

## Results

Sera from 215 cases and 650 controls were available for the study. The clinical signs most frequently presented by cases were: dizziness (58.1%); black vomitus and/or vomiting of blood (40.5%), and melena (19%). With regard to controls, most of patients were recruited from the pre-operative unit for minor surgical procedures: 295 (45.4%) for cataracts, and 168 (25.8%) for inguinal hernias. The demographic and clinical characteristics of cases and controls are listed in Table 1.

**Table 1:** Demographic, comorbid conditions and medication used in patients included in the case and control groups.

	Cases(n = 215)	Controls(n = 650)
**Demographics**		
Men, no.(%)	157 (73.0)	465 (71.5)
Age, mean (SD)	65.0 (±16.2)	63.4 (±15.6)
**Comorbid conditions (%)**		
Gastric ulcer*	40 (18.6)	45 (6.9)
Duodenal ulcer*	28 (13.0)	24 (3.7)
Unspecific ulcer*	0 (0.0)	3 (0.5)
Diabetes mellitus	27 (12.6)	89 (13.7)
Depression	38 (17.7)	112 (17.2)
Heart disease	62 (28.8)	133 (20.5)
Hypertension	81 (37.7)	234 (36.0)
Hypercholesterolemia	76 (35.3)	195 (30.0)
Arthrosis	59 (27.4)	190 (29.2)
Arthritis	16 (7.4)	40 (6.2)
Osteoporosis	11 (5.1)	31 (4.7)
Hepatic disease	12 (5.6)	59 (9.1)
*Helicobacter pylori*	201 (93.5)	549 (84.5)
Tobacco dependence	40 (18.6)	130 (20.0)
Alcohol		
No consumption	77 (35.8)	249 (38.3)
Low	83 (38.6)	269 (41.4)
Moderate	42 (19.5)	115 (17.7)
Heavy	13 (6.0)	17 (2.6)
**Medication**		
Drugs, Mean (SD)	3.90 (±2.6)	2.98 (±2.4)
NSAIDs, no. (%)	73 (34.0)	76 (11.7)
PPI, no. (%)	23 (10.7)	82 (12.6)
Antiplatelet drugs, no. (%)	50 (23.3)	94 (14.5)
Oral anticoagulants, no. (%)	19 (8.8)	35 (5.4)
Selective serotonin reuptake inhibitors, no. (%)	15 (7.0)	40 (6.2)

(*): Antecedent not directly related with current illness.

The results showed that 54 (25.1%) cases and 100 (15.4%) controls were positive for *Anisakis* (Ani s 1 or Ani s 7), while 73 (33.9%) cases and 76 (11.7%) controls were NSAID consumers (Table 2). Considering IgE determinations in cases plus controls, 85 subjects (55.2%) were seropositive for Ani 1 plus Ani s 7, 40 subjects (25.9%) positive for only Ani s 1, and 29 subjects (18.8%) positive for only Ani s 7. As regards cases, 30 (55.5%) patients were positive for both allergens, 16 (29.6%) patients were positive for only Ani s 1 and 8 (14.8%) patients were positive for only Ani s 7. In the control group 55 (55.0%) subjects were positive for both allergens, 24 (24.0%) subjects were positive for only Ani s 1 and 21 (21.0%) subjects were positive for only Ani s 7. With regard to sex distribution in *Anisakis* seropositive patients (either for Ani s 1 or Ani s 7 allergens), 35 cases (64.8%) and 76 controls (76.0%) were male.

**Table 2:** Odds ratios for upper gastrointestinal bleeding associated with NSAIDs and *Anisakis*, and their interaction.

Independent variables	Cases	Controls	AdjustedOR (95% CI)*	Synergistic Index (95% CI)
**MODEL 1: Model without interactions**				----
NSAIDs	73	76	5.80 (3.82–8.82)	
*Anisakis*	54	100	1.74 (1.13–2.69)	
**MODEL 2: Interaction between NSAIDs and ** ***Anisakis***				3.01 (1.18–7.71)
NSAID non-consumers				
*Anisakis*-negative	109	485	1.00	
*Anisakis*-positive	33	89	1.46 (0.87–2.43)	
NSAID consumers				
*Anisakis*-negative	52	65	5.01 (3.14–8.01)	
*Anisakis*-positive	21	11	14.45 (6.46–32.33)	

(*): Adjusted for past history of GI disorders, proton pump inhibitors, antiplatelet drugs, oral anticoagulants, reliability of interview.

To investigate the effects of the interaction between *Anisakis simplex* IgE sensitization and NSAID intake on risk of UGIB, we calculated the ORs values obtained for both variables with and without interactions. The results in Table 2, model 1, show the ORs values without interactions. *Anisakis* seropositive subjects registered a 1.74 fold higher risk of suffering from UGIB than seronegative subjects (95% CI: 1.13–2.69). However, when the effect of prior *Anisakis* infections was stratified by NSAID consumption (Table 2, model 2), we observed that this had no effect among non-consumers of NSAIDs (OR = 1.46, [95%CI: (0.87–2.43]), but that there was a more than 14-fold higher risk of UGIB (OR = 14.45 [95% CI: 6.46–32.33]) among NSAID consumers than in *Anisakis*-negative non-consumers of NSAIDs. The interaction was additive, with a synergistic index of 3.01 (95% CI: 1.18–7.71). Applying conditional logistic regression to these analyses provided very similar ORs, but with wider 95%CI range.

The data in Table 3 show the location and type of gastrointestinal lesions observed by endoscopy in cases, with respect to *Anisakis* sensitization and NSAIDs intake. There were no differences in the number and location of bleeding lesions between *Anisakis* sensitized patients, NSAIDs consumers, or patients lacking these risk factors. In addition, the endoscopic reports revealed a single case of acute anisakiasis. This corresponded to a 53-year-old female with three *Anisakis* larvae penetrating two gastric ulcers located in the fornix region of the stomach. The patient presented with hematemesis accompanied by dyspepsia and pyrosis, and was seropositive for Ani s 1 and Ani s 7 allergens.

**Table 3:** Type and location of endoscopic lesions observed in cases with respect to *Anisakis* sensitization and NSAIDs intake.

Type and location of lesions	*Anisakis*n = 54	NSAIDsn = 73	NSAIDs plus*Anisakis*n = 21	NSAIDs or*Anisakis*n = 85	Nonen = 109
**Erosions**					
Gastric	15 (27.8)	21 (28.8)	3 (14.3)	30 (35.3)	32 (29.4)
Duodenal	8 (14.8)	9 (12.3)	2 (9.5)	13 (15.3)	12 (11.0)
**Ulcers**					
Gastric	19 (35.2)	31 (42.4)	9 (42.9)	32 (37.6)	41 (37.6)
Duodenal	27 (50.0)	35 (47.9)	11 (52.4)	39 (45.9)	47 (43.1)

The numbers between parenthesis show the corresponding percentages.

## Discussion

This is the first epidemiological study showing that: i) prior *Anisakis* infections causing IgE sensitization are an independent risk factor for UGIB (with an almost twofold increase in the risk) and ii) that this effect is modified by NSAID consumption, to the extent that the risk of UGIB can increase by more than 14 times through a synergic effect between *Anisakis* and NSAIDs, showing in turn that the joint effect of the two risk factors is 3 times higher than the sum of their individual effects.

In the present study, a large number of subjects (15.4% in the control group) tested positive for Ani s 1 or Ani s 7 allergens. However, the results appear to be accurate because the combined Ani s 1 and Ani s 7 ELISAs used in this study are highly sensitive and specific in comparison with other serological methods [22]. The present results are also consistent with previous reports [15], [16] showing an extremely high seroprevalence for IgE antibodies to this parasite in the northern, central, and southern regions of Spain, where positive IgE values are observed in more than 10% of the population. Futhermore, other results showing that seropositive patients always had a prior history of ingestion of raw or undercooked fish [5] exclude the possibility that these high values were due to recognition of cross-reacting allergens present in other organisms such as mites [30]. *Anisakis* infections in Spain are mainly related to the ingestion of *boquerones en vinagre* ―pickled anchovies― [4], [5], although infections caused by eating undercooked fish (e.g. hake) have also been reported [4], [15], [31].

The presence of IgE antibodies in serum against specific secretory *Anisakis* allergens as Ani s 7, and probably Ani s 1, reveals that the patient has suffered one or more previous infections by this parasite [32]. However, for correct interpretation of the results, the effect of currently active and past *Anisakis* infections should be considered separately. In patients with active gastric anisakiasis, some of them may suffer erosions or hemorrhagic lesions of the mucosa, which can be detected by gastroscopy [4]. Bleeding during this phase can be explained by several causes, including: a) the marked inflammatory allergic status of the mucosa, accompanied by massive infiltration of eosinophils, neutrophils, macrophages and lymphocytes in response to parasite excretory antigens [33]; b) the direct erosive action of larvae moving into the gastric mucosa [34]; and c) the activity of proteases [35] and anticoagulant [36] substances released by the parasite.

In a recent study [22] we have observed that about 94% and 61% of symptomatic patients sensitized to *Anisakis* antigens have IgE antibodies to the Ani s 7 and Ani s 1 allergens, respectively. However, for patients that recognized both allergens the response to Ani s 1 was more prolonged in time. The data in the present study, showing that a considerable proportion of sera (25.9%) were only positive to the Ani s 1 allergen, suggest that many positive IgE results are due to past, unnoticed, *Anisakis* infections. In addition, it was reported that gastric *Anisakis* infections are much more frequent than duodenal anisakiasis [37]. The similar number of bleeding ulcerous lesions observed in our study at gastric and duodenal level, and the fact that only one positive case of active anisakiasis was detected by endoscopy, also suggest that the increased risk of UGIB in *Anisakis* seropositive patients is not due to active infections.

Unlike active anisakiasis, the implication of past *Anisakis* infections as a risk factor for UGIB is less evident. One could hypothesize, however, that effector molecules produced by defense cells previously activated in the GI tract in response to allergens and other *Anisakis* antigens, might provoke mucosal injury acting either alone or synergistically with other noxious factors present, such as NSAIDs. Candidate cells for mediating such action are eosinophils and, perhaps other pro-inflammatory cells that remain infiltrating the granulomatous tissue around the infecting larvae, or its debris, for long periods [37]. In particular, eosinophils are GI primary resident cells [38] which reportedly have immunoregulatory roles [39] and act as antigen-presenting cells in response to intestinal nematodes [40], and the cytotoxic preformed cationic proteins that they produce upon activation are able to cause mucosal damage, as seen in some intestinal inflammatory diseases [41], [42]. Eosinophils have also been reported to be present in the granulation tissue of perforated gastric ulcers in Japan, the country where *Anisakis* infections are most frequent, and the degree of infiltration by these cells was suggested to be a marker of perforation risk [43]. In this sense, it is thought that the matrix metalloproteinase-1 expressed in the cytoplasm of eosinophils may be able to digest collagen types I and III, which compose the stomach wall, and thus contribute to ulcer perforation [44]. Interestingly, NSAIDs also stimulate eosinophil production by downregulating PGE2 synthesis and upregulating production of cysteinyl leukotrienes [45] suggesting that the biochemical mechanisms whereby both risk factors potentiate UGIB may be interconnected.

As in *Anisakis* infections, it can be hypothesized that other infectious agents causing chronic infections of the upper gastrointestinal tract such as *Helicobacter pylori*, or food hypersensitivity [46], may also modify the risk of UGIB in NSAID consumers. In the present study, the observed synergism between NSAID consumption and prior *Anisakis* infections on the risk of UGIB were obtained from data adjusted by seroprevalence to *H. pylori*, thus discounting any possible bias caused by this confounding variable. Likewise, for food hypersensitivity to have a confounding effect there would have to be a positive correlation between food allergy and exposure to NSAIDs or infection by *Anisakis* larvae. However, this is not the case because there is no reason to think that subjects with food allergy may be more likely to consume NSAIDs or to be infected by the parasite.

Finally, it shoud be noted that because of the low prevalence of consumption of the individual NSAIDs in the sample, the individual effect of the interaction of each particular NSAID with *Anisakis* could not be observed. Nonetheless, since all NSAIDs share the same mechanism of action, it is expected that all act synergistically with *Anisakis* to a greater or lesser extent.

From the results of the present study we concluded that, in countries where there is a suspected presence of *Anisakis* infection, it would be wise to confirm whether or not the patient has a history of ingesting raw or undercooked fish before prescribing NSAIDs for long periods. For patients giving a positive response to this query, we recommend performing a parasite-specific IgE determination and conducting a closer follow-up during treatment with NSAIDs when the test is positive.
